# Unifying interdisciplinary education: designing and implementing an intern simulation educational curriculum to increase confidence in critical care from PGY1 to PGY2

**DOI:** 10.1186/s13104-017-2905-1

**Published:** 2017-11-06

**Authors:** Mark J. Bullard, Jo Anna Leuck, Lisa D. Howley

**Affiliations:** 1 Department of Emergency Medicine, Carolinas Simulation Center, Carolinas Medical Center, Carolinas HealthCare System, 1000 Blythe Blvd., 3rd Floor MEB, Charlotte, NC 28203 USA; 2 Department of Emergency Medicine, John Peter Smith Health System, Dallas, TX USA; 3 Association of American Medical Colleges, Washington, DC USA

**Keywords:** Critical care, Simulation, Communication, Confidence, Skills

## Abstract

**Background:**

A longitudinal, multidisciplinary critical care simulation curriculum was developed and implemented within a teaching hospital to address the need for consistent, safe, efficient, and unified critical care training within graduate medical education. Primary goals were to increase learner confidence in critical care topics and procedural skills across all specialties. Secondary goals included improving communication skills and obtaining a high level of learner satisfaction. All interns caring for adult patients within our hospital participated in three 4-h simulation-based sessions scheduled over the second half of their intern year. Pre- and postcurricular surveys evaluated self-confidence in critical care topics, procedures, and communication skills. The Debriefing Assessment for Simulation in Healthcare Student Version (DASH-SV) Short Form was used to evaluate facilitator debriefing. Data were compared with Wilcoxon rank sum and signed rank test.

**Results:**

Pre- and postcurricular surveys were collected from 51 of 52 interns (98% response rate) in curricular year 1 and 59 of 59 interns (100% response rate) in curricular year 2 in six programs within the hospital. Resident confidence significantly improved in all areas (*p* < .05). DASH-SV demonstrated overall effective facilitator debriefing and > 75% of interns in both curricular years 1 and 2 expressed a desire for future educational sessions.

**Conclusions:**

The implemented curriculum increased learner confidence in select critical care topics, procedures, and communication skills and demonstrated a high level of learner satisfaction. The curriculum has expanded to learners from three other teaching hospitals within our system to unify critical care education for all interns caring for adult patients.

**Electronic supplementary material:**

The online version of this article (10.1186/s13104-017-2905-1) contains supplementary material, which is available to authorized users.

## Background

Consistent, safe, efficient, and unified critical care training is needed within graduate medical education (GME). Increasing demands on physicians, resident work hour restrictions, increased patient acuity, and varying clinical experiences within GME have led to inconsistent exposure to topics and procedures crucial to preparing for intensive care unit (ICU) rotations. This apparent inconsistency in education has led residents to report being ill-prepared for ICU rotations [1].

The American College of Critical Care Medicine has recommended the use of simulation to enhance resident training in critical care [2]. Additionally, the Institute of Medicine report “To Err is Human” has recommended simulation training for physicians to reduce preventable errors [3]. Growing literature supports the use of simulation to educate and improve knowledge, skills, and attitudes in complex communication such as medical error disclosure [4] and death notification [5]. Within our hospital system, residency programs use simulation in variable capacities. Importantly, no overarching simulation curriculum exists for critical care emergencies and complex communication skills; rather, residents have varied exposure through bedside experiences.

To address these challenges, two emergency medicine faculty members trained in simulation debriefing and a behavioral psychologist developed and implemented a longitudinal, multidisciplinary critical care simulation curriculum. This program aimed to enhance and unify critical care education for all interns caring for adult patients within a tertiary care hospital system. The primary goal of the curriculum was to increase confidence in common critical care topics and select procedural skills. Secondary goals included improving confidence in communication skills and obtaining a high level of learner satisfaction.

## Methods

This novel simulation curriculum provided instruction on common critical care topics. Curricular design was based on a thorough needs assessment considering learner characteristics, institutional and system priorities, and time and space constraints. Curricular support was provided by a team of emergency medicine and critical care experts, ethicists, pastoral care, and experts in risk management.

Our learners were multi-specialty adult learners (internal medicine, general surgery, emergency medicine, family medicine, obstetrics/gynecology, and orthopedics) with busy clinical schedules and demands. As such, our curriculum focused on best practices in andragogy. Curricular design blended asynchronous, online, self-paced educational components with hands-on, reflective, and cooperative learning. Although learners had variable experience with simulation as an educational modality, simulation education was chosen for the curriculum due to its experiential and activating nature.

Topics and procedural skills imperative for all residents caring for ICU patients within our hospital were determined by a survey given to medical and surgical critical care colleagues and incorporated so that the curriculum would be applicable to all specialties. Included in these critical topic areas were important public health and system initiatives including the Surviving Sepsis campaign, target temperature management in postcardiac arrest patients, and expeditious use of thrombolytics in acute ischemic stroke care. Additionally, ethical and spiritual topics, such as medical error disclosure and death notification, were integrated to enhance communication skills perceived as lacking in residency education.

Each intern participated in three mandatory 4-h simulation-based sessions over a 6-month period. The curriculum began in January to avoid hospital orientation activities held in the first half of the educational year, to facilitate scheduling nearly 60 interns each year, and to allow appropriate time for interns to become comfortable with patient care requirements within their new hospital setting. Scheduling of simulation sessions was based on intern clinical duties, rotation call schedules, and resident work-hour restrictions. To avoid unexpected absenteeism, a designated scheduling coordinator reminded interns of their scheduled simulated sessions via e-mail as well as digital page the day before each session.

An introductory web-based module was developed for and delivered to all interns using a learning management system (MedHub, Inc., Ann Arbor, Michigan). This introduction provided an overview of the Laerdal Sim Man 3G platform (Laerdal Medical AS, Stavanger, Norway), reinforced principles of andragogy, and addressed curricular expectations, learning objectives, and the concept of “learner safety”.

Residents were presented with pre- and postsession education via the learning management system used in our residency program for independent, asynchronous review. Presession educational material included evidence-based literature on global themes related to the four cases covered during each session, clinical vignettes with case-based questions to encourage learners to begin thinking about topic-based clinical management, and hyperlinks to relevant procedural or topic videos. At the end of each session, educational material included more in-depth literature covering the same topics as well as a bulleted summary of topic objectives or “take-home points”. These bulleted points allowed learners to focus on group discussion rather than taking notes during the session while also providing facilitators the flexibility to focus specifically on topics perceived by the learners as most important to discuss. Asynchronous material introduced topics prior to the session to promote active group discussion, and concepts were reinforced at the end of each session.

Each simulation session consisted of four scenarios (Table 1) and was attended by four residents. Each scenario was led by one of the four residents, while the other three interns observed the scenario using real-time audio and visual feed. Immediately after each scenario, the scenario participant and the three observers were involved in a shared debriefing. All scenarios ran approximately 10–15 min and were designed as acute floor emergencies with the planned patient disposition to the ICU. In advanced cardiac life support (ACLS)-based scenarios (ventricular arrest, asystole, pulseless electrical activity arrest), one learner was designated the leader, while the others were active within the scenario as code members to add to the case fidelity. A nurse confederate was used to facilitate each simulated scenario, and scenarios concluded with appropriate consultation to the ICU. To increase realism and learner “buy-in,” care was taken to ensure that the background to each patient scenario or “stem” was applicable to the learner’s specialty. For example, the scenario of pulmonary embolism, led by an orthopedic intern, would involve a patient who was 3 weeks postoperative from external fixation of a fracture; if this scenario was led by a gynecology intern, it would involve a patient of posthysterectomy status. During scenarios focusing on communication skills (death notification and medical error disclosure), a standardized participant acted as a simulated family member. Finally, to create experiential fidelity in cerebral vascular accidents, a standardized patient was used to demonstrate acute hemiplegia and aphasia. For procedural skills, task trainers were incorporated within the context of the case as well as outside the direct scenario for additional deliberate practice.

**Table 1:** Overview of simulation sessions

Session #1	
ACLS—ventricular tachycardia/therapeutic hypothermia	
Anaphylaxis/medication error disclosure	
Central line/ultrasound guided central line placement	
Surviving sepsis campaign	
Session #2	
ACLS—asystole/death notification	
Symptomatic bradycardia-transcutaneous pacing	
Pulmonary embolism	
ACS/NSTEMI	
Session #3	
ACLS—unstable SVT/synchronized cardioversion	
Status epilepticus/lumbar puncture	
PEA/hyperkalemia/airway management (video laryngoscopy)	
Cerebral vascular accidents and thrombolytics	

Following each scenario, a shared debriefing consisting of two faculty, the scenario participant, and three observers to the scenario took place. Debriefing lasted approximately 45 min and explored immediate reactions, course objectives, clinical management, pathophysiology, procedural instruction, and guided feedback to improve future clinical performance. Debriefing was facilitated by faculty members specifically trained in simulation education who allowed the learners to explore their learning needs while insuring curricular objectives were met. Additionally, domain experts from pastoral care, ethics, and risk management co-debriefed during scenarios involving medical error disclosure and death notification.

Resident performance was evaluated via summative, norm-referenced assessment and was shared with residency program directors at the conclusion of the curriculum to help identify residents in need of further education or oversight prior to or during their PGY-2 ICU rotations. Additional simulation opportunities were built into the curriculum for any learner identified as needing remediation.

For continued quality improvement of the curriculum, learners were surveyed on primary and secondary goals immediately prior to and upon completion of the curriculum (Additional file 1: Appendix S1). Confidence-based questions were measured on a 4-point Likert scale ranging from 0 (not at all confident) to 3 (very confident). Additionally, each debriefing was evaluated using the Debriefing Assessment for Simulation in Healthcare Student Version (DASH-SV) [6] Short Form to improve and ensure that debriefing was of the highest quality. The Wilcoxon rank sum and signed rank tests were used to compare these data and corresponding 95% confidence intervals for the difference in means were reported. SAS^®^, version 9.3 (SAS Institute, Inc., Cary, North Carolina) was used for all analyses, and a *p* value < .05 was considered significant. While the curriculum was mandatory for all interns caring for adult patients, all data were voluntarily collected as part of a curricular evaluation process, and retrospective review of the data was approved by the hospital Institutional Review Board under waiver of informed consent. Surveys were administered during the simulation sessions with a capture rate of nearly 100%.

## Results

Pre- and postcurricular surveys were collected from 51/52 interns (curricular year 1; 98% response rate) and 59/59 interns (curricular year 2; 100% response rate) from six residency programs involved in the curriculum.

Curricular data demonstrated significant increases (*p* < .05) in resident confidence in critical care topics as well as procedural and communication skills (Table 2). Learner confidence in the treatment of anaphylaxis, sepsis, acute coronary syndromes, status epilepticus, pulmonary embolism, ACLS algorithms, and cerebral vascular events all increased with significance. Additionally, with exception to endotracheal intubation for curricular year 1, confidence in procedural skills (defibrillation, transcutaneous pacing, cardioversion, central line placement, lumbar puncture) and communication skills (medical error notification and death notification) increased with significance in both curricular years 1 and 2. Finally, more than 75% of interns in both curricular years 1 and 2 stated a desire to attend future sessions.

**Table 2:** Mean learner confidence pre- and postcurriculum in year 1 (*n* = 51) and year 2 (*n* = 59)

Curricular topic	Pre	Post	*p* value	95% CI
Anaphylaxis				
Year 1	0.73	1.57	< .0001	(0.60, 1.09)
Year 2	0.78	1.73	< .0001	(0.78, 1.22)
Sepsis				
Year 1	1.00	1.84	< .0001	(0.59, 1.09)
Year 2	1.00	2.03	< .0001	(0.81, 1.30)
Acute coronary syndrome				
Year 1	1.02	1.65	< .0001	(0.38, 0.88)
Year 2	0.88	1.82	< .0001	(0.73, 1.13)
Status epilepticus				
Year 1	0.67	1.63	< .0001	(0.70, 1.21)
Year 2	0.61	1.73	< .0001	(0.90, 1.38)
Pulmonary embolism				
Year 1	1.19	1.80	< .0001	(0.34, 0.88)
Year 2	1.07	1.95	< .0001	(0.64, 1.15)
Advanced cardiac life support				
Year 1	0.96	1.75	< .0001	(0.56, 1.01)
Year 2	0.81	1.88	< .0001	(0.87, 1.31)
Cerebral vascular accident				
Year 1	1.00	1.59	< .0001	(0.34, 0.83)
Year 2	0.92	1.87	< .0001	(0.71, 1.25)
Endotracheal intubation				
Year 1	0.98	1.24	.1510	(− 0.07, 0.58)
Year 2	0.78	1.33	< .0001	(0.30,0 .86)
Central line placement				
Year 1	1.13	1.76	.0006	(0.30, 0.96)
Year 2	1.02	1.55	.0003	(0.26, 0.86)
Lumbar puncture				
Year 1	1.12	1.63	.0041	(0.19, 0.84)
Year 2	0.98	1.52	.0003	(0.27, 0.89)
Defibrillation				
Year 1	0.75	1.47	< .0001	(0.43, 1.01)
Year 2	0.66	1.63	< .0001	(0.70, 1.23)
Transcutaneous pacing				
Year 1	0.38	1.08	< .0001	(0.43, 0.95)
Year 2	0.36	1.32	< .0001	(0.72, 1.17)
Cardioversion				
Year 1	0.42	1.22	< .0001	(0.53, 1.06)
Year 2	0.41	1.48	< .0001	(0.81, 1.30)
Death notification				
Year 1	0.96	1.82	< .0001	(0.62, 1.10)
Year 2	0.98	1.88	< .0001	(0.62, 1.13)
Medical error notification				
Year 1	0.85	1.63	< .0001	(0.53, 1.04)
Year 2	1.00	1.75	< .0001	(0.50, 1.01)

All data are represented as mean ± standard deviation

*ACLS* advanced cardiac life support, *CI* confidence interval

The DASH-SV results demonstrated effective facilitator debriefing (Table 3). In each of the areas scored by learners, the facilitators averaged scores of > 6, which corresponded to “consistently effective debriefing” in both curricular years 1 and 2. All debriefing domains measured by the DASH-SV demonstrated improvements from curricular year 1–2: faculty set stage for engaging learning experience, facilitators maintained an engaging context for learning, instructor structured debriefing in an organized way, facilitators provoked in-depth discussions that led me to reflect on my performance, instructors identified what I did well or poorly, and instructors helped me see how to improve or sustain good performance, with all but one domain demonstrating statistical significance.

**Table 3:** Debriefing assessment for simulation in healthcare student version (DASH-SV) scores

Aspect evaluated	Year 1	Year 2	*p* value	95% CI
Faculty set stage for engaging learning experience	6.47	6.56	.0793	− 0.22, 0.04
Facilitators maintained an engaging context for learning	6.50	6.61	.0483	− 0.24, − 0.02
Instructor structured debriefing in an organized way	6.39	6.58	.0053	− 0.32, − 0.05
Facilitators provoked in-depth discussions that led me to reflect on my performance	6.39	6.61	.0021	− 0.36, − 0.08
Instructors identified what I did well or poorly	6.05	6.52	< .0001	− 0.63, − 0.31
Instructors helped me see how to improve or sustain good performance	6.26	6.54	< .0001	− 0.42, − 0.14

Data are presented as mean ± standard deviation

*CI* confidence interval

## Discussion

Medical simulation affords practice for low frequency/high acuity scenarios, procedural skills prior to a patient encounter, and complex ethical and spiritual communication. Also, simulation education provides a forum for interdisciplinary education: to learn and practice important skill sets and communication skills while training together. By supplying “education on demand,” simulation alleviates many educational constraints while unifying resident exposure to critical thinking, procedural skills, and important interpersonal professional skill sets. This curriculum was developed, implemented, and successfully improved learner confidence in critical care topics, procedural skills, and communication skills while creating a curriculum with a high level of learner satisfaction.

Our system demonstrated a need for such a curriculum. As previously cited in the literature, inconsistencies in exposure and confidence in critical care was validated by an internal survey of residents within our hospital system 6 months into their intern year. This survey demonstrated that nearly half of our interns did not feel properly prepared to care for critically ill patients and that experience and confidence in critical care topics, resuscitation, procedural skills, and communication skills were also shown to be limited. While increasing learner confidence in select critical care topics, procedural skills, and communication skills confidence, this curriculum also addressed a common problem, also validated by our survey, of training physicians having limited direct supervision and feedback by attending physicians.

The DASH is widely known as an evidence-based debriefing assessment tool showing validity, reliability, and feasibility [7]. The DASH-SV results demonstrated effective facilitator debriefing (Table 3). In each of the areas scored by learners, the facilitators averaged scores of > 6, which corresponded to “consistently effective debriefing” in both curricular years 1 and 2. After reviewing curricular year 1 DASH-SV data, the facilitators made concerted efforts to improve debriefing behaviors in each of these areas, and DASH-SV scores in curricular year 2 reflected adjustments in debriefing, and consequently improvements in the learning environment.

This study has limitations, including being limited by a short timeframe (6 months) of learner review over 2 curricular year cycles. Additionally, this study unfortunately did not aim to measure intern knowledge acquisition, and/or account for any education or experiences on curricular topics external to that of the 6-month simulation curriculum. This may have influenced learner confidence.

Whether increased confidence equates to increased knowledge acquisition remains unknown; however, open-ended survey feedback showed considerable knowledge acquisition and improvement in real clinical practice. Multiple residents reported encounters with very similar critically ill patients within the hospital and noted the direct benefits of knowledge and confidence gleaned from this experiential curriculum in caring for these patients. Unfortunately, this educational intervention and study was designed to increase learner confidence in critical care topics, and data on knowledge acquisition was not collected. This, however, creates opportunity for future work to better define simulation-based knowledge acquisition for critical care events within this annual curriculum.

It is impossible to state that increased learner confidence is directly correlated to the curriculum, as the authors did not make efforts to identify or quantify confounding education or experiences on these critical topic areas during the study period. This said, this curriculum is one of the only educational experiences that allows all six specialties within our hospital system to focus on critical care skill sets that reside outside specific daily education and specialty domains.

## Conclusions

Using medical simulation technology, a novel, longitudinal, multidisciplinary critical care simulation curriculum for all interns caring for adult patients was developed and implemented within a tertiary care hospital system. This experiential curriculum resulted in increased learner confidence in select critical care topics, procedural skills, and communication skills prior to matriculation to the second postgraduate year. This curriculum resulted in a high level of learner satisfaction and desire for additional simulation-based training within their second postgraduate year. Due to the success of this local curricular innovation, the curriculum has expanded to learners from three other teaching hospitals within our system and has unified critical care education for all interns caring for adult patients at multiple hospitals within our system.

## Additional file

**Additional file 1: Appendix S1.** Pre/post curricular survey.

## Abbreviations

DASH-SV: Debriefing Assessment for Simulation in Healthcare Student Version Short Form
ICU: intensive care unit
GME: graduate medical education
ACLS: advanced cardiac life support
PGY-2: post graduate year 2
IRB: institutional review board
