# Supplementary material for: Unifying interdisciplinary education: designing and implementing an intern simulation educational curriculum to increase confidence in critical care from PGY1 to PGY2
Source: BMC Res Notes. 2017 Nov 6;10:563. doi: 10.1186/s13104-017-2905-1 (PMC5674682; doi:10.1186/s13104-017-2905-1)
Supplement: Supplementary file 1 — Additional file 1: Appendix S1. Pre/post curricular survey. [file 13104_2017_2905_MOESM1_ESM.doc]

Question 1 - Choice - One Answer (Bullets)

How many times have you been the 'leader' of a critical medical resuscitation?

- 0
- 1-2
- 3-4
- 5-6
- 7-8
- 9-10
- 11+

Page 1 - Question 2 - Choice - One Answer (Bullets)

Do you feel that your training (to date) has prepared you to care for critically ill patients?

- Not At All
- Very Little
- Somewhat
- To A Great Extent

Question 3 - Rating Scale

Approximately how many times have you been involved with delivery of care for patients suffering from the following conditions during medical school and/or residency? Select the circle corresponding to the number or range which best reflects your estimate.

|  | 0 | 1-5 | 6-10 | 11-15 | 16-20 | 21-25 | 26-30 | 30+ |
| --- | --- | --- | --- | --- | --- | --- | --- | --- |
| Anaphylactic Shock | O | O | O | O | O | O | O | O |
| Septic Shock | O | O | O | O | O | O | O | O |
| Acute Coronary Syndrome | O | O | O | O | O | O | O | O |
| Status Epilepticus | O | O | O | O | O | O | O | O |
| Pulmonary Embolism | O | O | O | O | O | O | O | O |
| Cardiac Arrhythmias/ACLS | O | O | O | O | O | O | O | O |
| Cerebral Vascular Accidents | O | O | O | O | O | O | O | O |

Question 4 - Rating Scale

Please rate your level of confidence caring for the following conditions competently with a live patient - with minimal or no supervision. Select the circle corresponding to the number or range which best reflects your estimate.

|  | Not At All | Somewhat | Confident | Very Confident |
| --- | --- | --- | --- | --- |
| Anaphylactic Shock | O | O | O | O |
| Septic Shock | O | O | O | O |
| Acute Coronary Syndrome | O | O | O | O |
| Status Epilepticus | O | O | O | O |
| Pulmonary Embolism | O | O | O | O |
| Cardiac Arrhythmias/ACLS | O | O | O | O |
| Cerebral Vascular Accidents | O | O | O | O |

Question 5 - Rating Scale

Approximately how many times have you performed the following procedures on a live patient during medical school and/or residency? Select the circle corresponding to the number or range which best reflects your estimate.

|  | 0 | 1-5 | 6-10 | 11-15 | 16-20 | 21-25 | 26-30 | 30+ |
| --- | --- | --- | --- | --- | --- | --- | --- | --- |
| Central Line Placement | O | O | O | O | O | O | O | O |
| Endotracheal Intubation | O | O | O | O | O | O | O | O |
| Lumbar Puncture | O | O | O | O | O | O | O | O |
| Defibrillation | O | O | O | O | O | O | O | O |
| Transcutaneous Pacing | O | O | O | O | O | O | O | O |
| Cardioversion | O | O | O | O | O | O | O | O |

Question 6 Rating Scale

Approximately how many times have you been DIRECTLY OBSERVED by a senior physician (faculty member or senior resident) while performing the following procedures on a live AND/OR simulated patient during medical school and/or residency? Select the circle corresponding to the number or range which best reflects your estimate.

|  | 0 | 1-5 | 6-10 | 11-15 | 16-20 | 21-25 | 26-30 | 30+ |
| --- | --- | --- | --- | --- | --- | --- | --- | --- |
| Central Line Placement | O | O | O | O | O | O | O | O |
| Endotracheal Intubation | O | O | O | O | O | O | O | O |
| Lumbar Puncture | O | O | O | O | O | O | O | O |
| Defibrillation | O | O | O | O | O | O | O | O |
| Transcutaneous Pacing | O | O | O | O | O | O | O | O |
| Cardioversion | O | O | O | O | O | O | O | O |

Question 7 - Rating Scale

Please rate your level of confidence performing the following procedures on a live patient - with minimal or no supervision. Select the descriptor which best reflects your estimate.

|  | Not At All | Somewhat | Confident | Very Confident |
| --- | --- | --- | --- | --- |
| Central Line Placement | O | O | O | O |
| Endotracheal Intubation | O | O | O | O |
| Lumbar Puncture | O | O | O | O |
| Defibrillation | O | O | O | O |
| Transcutaneous Pacing | O | O | O | O |
| Cardioversion | O | O | O | O |

Question 8 - Rating Scale

Approximately how many times have you done the following? Select the number or range which best represents your estimate.

|  | 0 | 1-5 | 6-10 | 11-15 | 16-20 | 21-25 | 26-30 | 30+ |
| --- | --- | --- | --- | --- | --- | --- | --- | --- |
| Delivered Death Notification to Family Member/Love One of Patient? | O | O | O | O | O | O | O | O |
| Notified a Patient, Family Member or Loved One of a Medical Error? | O | O | O | O | O | O | O | O |

Question 9 - Rating Scale

Please rate your level of confidence performing the following. Select the descriptor which best reflects your estimate.

|  | Not At All | Somewhat | Confident | Very Confident |
| --- | --- | --- | --- | --- |
| Death Notification | O | O | O | O |
| Delivering News of Medical Error | O | O | O | O |

Question 10 - Open Ended - Comments Box

Please use the space below to comment on any of the above.
